# Fine-Scale Temperature-Dependent Shifts in Lactic Acid Bacterial Communities Under Precise Peltier Control

**DOI:** 10.3390/microorganisms14071457

**Published:** 2026-07-02

**Authors:** Jin-Hee Seo, Kyung June Yim, Ji-Yeon Chun, Hye-Yoon Yi, Mi-Ju Kim, Hae-Won Lee

**Affiliations:** 1 Department of Food Bioengineering, Jeju National University, Jeju 63243, Republic of Korea; wlsgml6318@naver.com (J.-H.S.); kj.yim2012@gmail.com (K.J.Y.); chunjiyeon@jejunu.ac.kr (J.-Y.C.); yiyh88@jejunu.ac.kr (H.-Y.Y.); 2 Food Tech Center (FTC), Jeju National University, Jeju 63243, Republic of Korea; 3 Department of Food Science and Biotechnology, Kyung Hee University, Yongin 17104, Republic of Korea; mijukim@khu.ac.kr; 4 Research Institute of Advanced Technology, Jeju National University, Jeju 63243, Republic of Korea

**Keywords:** lactic acid bacteria, temperature sensitivity, Peltier thermocycler, microbial community, 16S rRNA sequencing

## Abstract

Temperature is a key factor shaping microbial growth and community structure, but the effects of fine-scale temperature differences remain insufficiently characterized. A Peltier-based PCR thermocycler was used as a precise micro-incubation platform (±0.1 °C) to evaluate responses of a commercial lactic acid bacterial starter culture at 1 °C intervals. Starter suspensions were incubated at 3, 4, 5, 36, 37, and 38 °C for 48 h, and species-level community composition was assessed by V3–V4 16S rRNA gene sequencing. Low-temperature conditions produced stable communities, whereas high-temperature conditions induced significant species-level differences among 36–38 °C groups, particularly for *Limosilactobacillus reuteri*, *Limosilactobacillus fermentum*, *Lactobacillus helveticus*, and *Lactiplantibacillus plantarum* (FDR < 0.01). At 37 °C versus 38 °C, the relative abundance of *Limosilactobacillus reuteri* increased from 4.56% to 9.31% (a 2.04-fold change), while the relative abundance of *Limosilactobacillus fermentum* decreased from 64.00% to 48.58%. These results highlight condition-dependent temperature sensitivity in lactic acid bacterial communities, with compositional responses differing markedly between the cold (3–5 °C) and warm (36–38 °C) ranges tested.

## 1. Introduction

Temperature is one of the most important environmental factors controlling microbial cultivation, growth kinetics, metabolic activity, and community composition. Lactic acid bacteria exhibit temperature-dependent growth behavior, and previous studies have shown that growth rate can increase markedly as incubation temperature approaches the optimal range [1,2]. Additionally, even small temperature variations can significantly influence microbial behaviour, survival, and ecological functions, as temperature affects the rate of nearly all biochemical reactions within cells [3]. However, conventional incubators may show spatial gradients and delayed responses during temperature regulation, which can obscure subtle biological responses to small thermal differences [4].

PCR instruments are designed to regulate reaction temperatures rapidly and accurately during thermal cycling. This precise control is generally achieved using thermoelectric modules based on the Peltier effect [5,6]. Unlike conventional incubators or water baths, which are generally limited to coarser temperature control and can exhibit spatial temperature gradients across the incubation chamber [4], Peltier-based thermocyclers provide spatially uniform, programmable heating and cooling with sub-degree precision. The Applied Biosystems SimpliAmp™ Thermal Cycler used in this study offers a stated temperature control precision of ±0.1 °C, enabling discrete 1 °C step increments to be applied simultaneously to multiple replicate samples under highly reproducible conditions. Although such devices are primarily used for nucleic acid amplification, their small reaction chambers and accurate thermal control make them suitable as micro-incubation platforms for testing fine-scale microbial temperature responses [7].

Most studies on bacterial temperature responses have focused on broad temperature ranges or growth-rate changes. In contrast, the effects of narrow temperature intervals, such as 1 °C differences, on microbial community structure remain poorly understood. These fine-scale responses may be especially relevant for starter cultures and fermentation systems, where small temperature variations can influence species competitiveness without necessarily changing total biomass. Therefore, this study used a Peltier-based thermocycler to evaluate temperature-sensitive shifts in lactic acid bacterial communities at 1 °C intervals in low- and high-temperature ranges.

## 2. Materials and Methods

### 2.1. Starter Culture Preparation and Peltier-Controlled Incubation

One sachet of a commercial lactic acid bacterial starter (Viqueen, Seoul, Republic of Korea) was inoculated into 100 mL of sterile de Man, Rogosa and Sharpe (MRS) broth (MBcell, KisanBio, Seoul, Republic of Korea) and thoroughly homogenized using a magnetic stirrer or vortex mixer to prepare a master inoculum. According to the manufacturer-provided product information, the starter contains SACCO-derived six lactic acid bacterial species and CENTRO-derived 17 mixed lactic acid bacterial species, with a declared total of approximately 1.3 × 10^12^ viable lactic acid bacteria. Species-specific or strain-specific CFU values were not provided by the manufacturer. From this suspension, 1 mL was aseptically transferred into 9 mL of sterile MRS broth to obtain a 10^−1^ diluted working inoculum, which was applied uniformly across all experimental conditions. The working inoculum was dispensed into sterile 0.2 mL PCR tubes (100 µL per tube), with 16 tubes prepared for each temperature condition. Incubation was performed without agitation for 48 h at 3, 4, 5, 36, 37, and 38 °C (±0.1 °C) using an Applied Biosystems SimpliAmp™ Thermal Cycler (Thermo Fisher Scientific, Waltham, MA, USA). For each temperature condition, the 16 PCR tubes were pooled into a single composite sample prior to DNA extraction and downstream community analysis. The pooled sample was then subjected to triplicate analysis, and these three measurements were used as analytical replicates. These replicates do not represent fully independent biological replicates, as pooling occurred prior to DNA extraction.

### 2.2. 16S rRNA Gene Sequencing and Bioinformatics

After incubation, DNA/RNA Shield was added to each sample at twice the sample volume, followed by vortexing and brief centrifugation to ensure homogenization. The treated samples were stored at 4 °C until microbial community analysis. The V3–V4 regions of the bacterial 16S rRNA gene were amplified using region-specific fusion primers, generating amplicons of approximately 500–700 bp. Sequencing libraries were constructed from the amplified products and sequenced on an Illumina MiSeq platform (2 × 250 bp paired-end reads; Illumina, San Diego, CA, USA). Low-quality reads were filtered using Trimmomatic v0.32 [8], paired-end reads were merged and dereplicated using VSEARCH v2.13.4 [9], and non-specific amplicons were identified using nhmmer in HMMER v3.2.1 [10]. Taxonomic assignment was conducted against the EzBioCloud 16S rRNA database [11] using VSEARCH, chimeric sequences were removed using UCHIME, and additional operational taxonomic units were generated through de novo clustering. Subsequent statistical analyses were performed using MicrobiomeAnalyst 2.0 [12]. Because species-level assignment was based on V3–V4 16S rRNA amplicon sequences, closely related lactic acid bacterial taxa should be interpreted cautiously, particularly taxa within recently reclassified Lactobacillus-related genera. Species-level names are therefore used as database-based taxonomic assignments rather than strain-resolved identifications.

### 2.3. Viable Cell Count Assay

To assess whether adjacent high-temperature conditions affected viable counts independently of community composition, *Limosilactobacillus fermentum* BIOBALL^®^ (bioMérieux, Marcy-l’Étoile, France) was used as a standardized culture. The BIOBALL was dissolved in MRS broth, and cultures were incubated at 36, 37, and 38 °C for 48 h. After incubation, serial dilutions were prepared using sterile MRS broth, and 100 µL of the appropriate dilution was spread onto MRS agar plates. Plates were incubated at 37 °C for 24 h, and plates containing 30–300 colonies were selected. Viable cells were expressed as colony-forming units per milliliter (CFU/mL). All experiments were performed in triplicate, and results are presented as mean ± standard deviation (SD).

### 2.4. API ZYM Enzymatic Activity Assay

Enzymatic activity profiles of the bacterial starter cultures were determined using the API ZYM system (bioMérieux, Marcy-l’Étoile, France). Starter cultures were incubated at 37 and 38 °C for 48 h using the PCR-tube incubation setup described above. For each temperature condition, 32 PCR tubes were prepared and pooled after incubation. Pooled cultures were centrifuged at 10,000× *g* for 5 min at room temperature, washed with sterile 0.85% NaCl, and resuspended in sterile 0.85% NaCl. Turbidity was adjusted to approximately McFarland 5–6 before inoculation. For each API ZYM cupule, 65 µL of the bacterial suspension was added, and strips were incubated at 37 °C for 4 h. ZYM A and ZYM B reagents were applied sequentially, and color development was evaluated using the manufacturer’s color chart. Scores ranged from 0 to 5, and scores of ≥3 were considered positive.

### 2.5. Statistical Analysis

Taxonomic composition at the species level was visualized using stacked bar plots based on relative abundance. Differences among temperature groups were evaluated using one-way analysis of variance (ANOVA), and *p*-values were adjusted for multiple comparisons using the false discovery rate (FDR) method. Accordingly, the ANOVA/FDR results should be interpreted as exploratory evidence of temperature-associated compositional differences rather than definitive differential-abundance results. In addition, group differences with adjusted *p*-values of <0.05 were considered statistically significant. Viable cell counts were also evaluated by one-way ANOVA.

## 3. Results

### 3.1. Temperature-Dependent Community Composition

**Figure 1:**
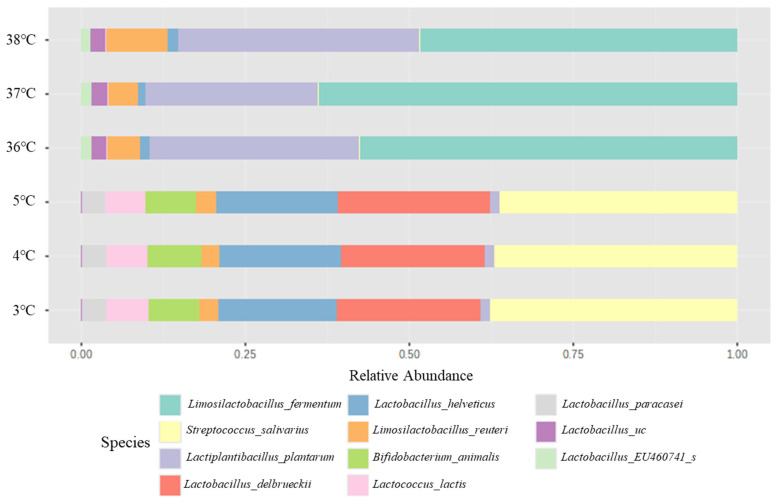
Relative species-level composition of lactic acid bacterial communities incubated at different temperatures. Stacked bar plots show the relative abundance of bacterial species at 3, 4, 5, 36, 37, and 38 °C. At 36–38 °C, *Limosilactobacillus fermentum* was the predominant species. At 3–5 °C, other species, including *Lactiplantibacillus plantarum*, *Streptococcus salivarius*, and *Lactobacillus delbrueckii*, represented substantial proportions of the community.

Species-level analysis revealed distinct compositional patterns between low-temperature (3–5 °C) and high-temperature (36–38 °C) conditions (Figure 1). Low-temperature groups showed a complex and balanced composition, including *Streptococcus salivarius*, *Lactobacillus delbrueckii*, *Lactobacillus helveticus*, *Bifidobacterium animalis*, *Lactococcus lactis*, and *Lactobacillus paracasei*. In contrast, high-temperature groups showed a simplified structure characterized by marked enrichment of *Limosilactobacillus fermentum* and other Lactobacillus-related taxa. Notably, *Limosilactobacillus fermentum* was nearly absent under low-temperature conditions, consistent with previous reports indicating that some *Limosilactobacillus fermentum* strains fail to grow at low temperatures [13].

**Figure 2:**
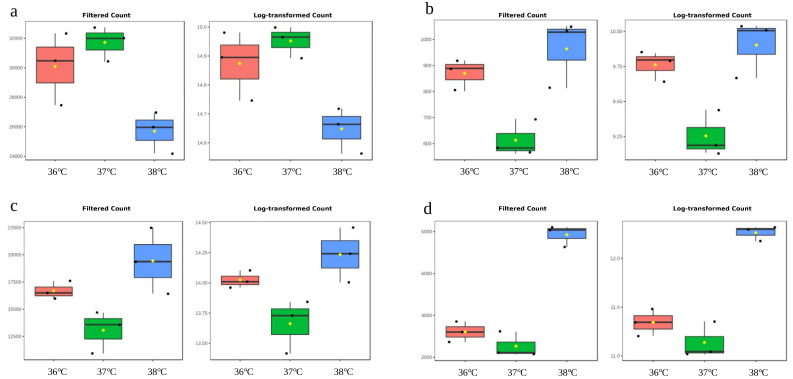
Temperature-dependent changes in significantly altered taxa within 36–38 °C. Filtered and log-transformed counts of Limosilactobacillus fermentum (**a**), Lactobacillus helveticus (**b**), Lactiplantibacillus plantarum (**c**), and Limosilactobacillus reuteri (**d**) across 36 °C, 37 °C, and 38 °C. Differences were assessed by one-way ANOVA with false discovery rate (FDR) correction applied to analytical replicates. FDR values were 0.00032 for Limosilactobacillus reuteri, 0.00274 for Limosilactobacillus fermentum, 0.00557 for Lactobacillus helveticus, and 0.00748 for Lactiplantibacillus plantarum; all four taxa met the significance threshold of FDR < 0.01. Statistical significance is indicated by the reported FDR-adjusted values rather than by asterisks.

Within the low-temperature range, one-way ANOVA with FDR correction applied to analytical replicates detected no statistically significant species-level differences among the 3, 4, and 5 °C groups (all FDR > 0.05), indicating that 1 °C intervals did not induce detectable compositional shifts under these conditions. By contrast, in the high-temperature range, one-way ANOVA applied to analytical replicates indicated differences among the 36, 37, and 38 °C groups for *Limosilactobacillus reuteri* (FDR = 0.00032), *Limosilactobacillus fermentum* (FDR = 0.00274), *Lactobacillus helveticus* (FDR = 0.00557), and *Lactiplantibacillus plantarum* (FDR = 0.00748) (Figure 2). These findings suggest that microbial community responses to fine-scale temperature differences differ between the cold (3–5 °C) and warm (36–38 °C) temperature conditions tested, rather than being uniform across temperature ranges [14]. Because growth rate increases sharply as incubation temperature approaches the optimum but remains low and relatively insensitive to small changes near the lower growth boundary, the magnitude of compositional response to 1 °C increments is expected to differ between the cold (3–5 °C) and warm (36–38 °C) ranges examined here; mapping this response across intermediate temperatures (e.g., 10–35 °C) remains an important objective for future work [1].

### 3.2. Viable Cell Counts at Adjacent High Temperatures

To determine whether the observed species-level compositional differences were accompanied by changes in viable counts, a standardized *L. fermentum* culture was incubated at 36, 37, and 38 °C. Viable cell counts were 9.03 ± 0.07, 9.05 ± 0.04, and 9.05 ± 0.01 log CFU/mL at 36, 37, and 38 °C, respectively (Table 1). No significant differences were detected among the temperature conditions (one-way ANOVA, F = 0.277, *p* = 0.767). These results indicate that *Limosilactobacillus fermentum* maintained comparable viable counts across 36–38 °C under single-culture conditions. This finding contrasts with the 16S rRNA sequencing results, in which the relative abundance of *Limosilactobacillus fermentum* differed among the same temperature conditions. The discrepancy suggests that the observed changes in *Limosilactobacillus fermentum* abundance in the starter culture were not driven simply by direct temperature effects on *Limosilactobacillus fermentum* viability. Rather, they may reflect shifts in the competitive balance among community members, potentially involving temperature-dependent responses of coexisting lactic acid bacteria and interspecies interactions [14,15].

### 3.3. API ZYM Enzymatic Activity Profiles

API ZYM analysis showed highly similar enzymatic activity profiles between starter cultures incubated at 37 and 38 °C for 48 h (Table 2). Positive reactions were observed for leucine arylamidase, valine arylamidase, acid phosphatase, naphthol-AS-BI-phosphohydrolase, α-galactosidase, β-glucuronidase, α-glucosidase, and β-glucosidase under both conditions. Other enzymatic reactions were weak or negative. These data indicate that the 1 °C difference between 37 and 38 °C did not substantially change the broad semi-quantitative enzyme activity profile of the starter culture, despite species-level compositional differences detected by sequencing.

## 4. Discussion

The present study demonstrates that fine-scale temperature differences can affect lactic acid bacterial communities in a condition-dependent manner, with compositional responses differing markedly between the cold (3–5 °C) and warm (36–38 °C) temperature conditions tested. The absence of detectable variation within 3–5 °C suggests that the low-temperature range imposed a broad constraint on community dynamics, possibly limiting metabolic activity and competitive differentiation. Conversely, compositional differences among 36–38 °C conditions suggest that communities near favorable or upper growth ranges may respond more sensitively to small thermal changes.

The lack of significant differences in *L. fermentum* viable counts across 36–38 °C indicates that the observed community changes were not simply explained by direct effects of temperature on *L. fermentum* viability under single-culture conditions. Because the absolute viable counts of *L. fermentum* remained stable across 36–38 °C, the corresponding shift in its relative abundance more likely reflects temperature-dependent changes in the abundance of co-occurring taxa, potentially involving interspecies interactions among coexisting lactic acid bacteria [14,15]. This interpretation is consistent with previous observations that temperature can structure microbial communities at broad scales [16] and that microbial interactions are sensitive to small environmental changes [14].

API ZYM profiles were also similar between 37 and 38 °C, indicating that broad enzymatic activity patterns were relatively stable despite compositional shifts. In practical terms, this suggests that even small, unintended deviations in storage or fermentation temperature (on the order of 1 °C) could meaningfully alter the species composition of LAB starter cultures, with potential downstream effects on product flavor, texture, and consistency, underscoring the value of precise temperature control during fermentation and cold-chain storage. The eight enzymes scored as positive (≥3) at both 37 °C and 38 °C in Table 2—leucine arylamidase, valine arylamidase, acid phosphatase, naphthol-AS-BI-phosphohydrolase, α-galactosidase, β-glucuronidase, α-glucosidase, and β-glucosidase—remained unchanged despite the compositional shift among *Limosilactobacillus reuteri*, *Limosilactobacillus fermentum*, *Lactobacillus helveticus*, and *Lactiplantibacillus plantarum*, consistent with functional redundancy among co-occurring lactic acid bacteria for these activities [15]. This finding suggests that community composition may represent a more sensitive indicator of fine-scale thermal effects than total viable counts or semi-quantitative enzyme profiles. However, this study was limited to a commercial starter culture and selected temperature ranges. Furthermore, because the 16S rRNA sequencing data were generated from pooled analytical replicates rather than independently processed biological replicates, the statistical interpretation should be made cautiously. Future studies using independently processed biological replicates and absolute quantification methods, such as qPCR, will be necessary to confirm which specific taxa are responsible for the observed compositional shifts.

Overall, a Peltier-based PCR thermocycler provided a simple and reproducible micro-incubation platform for assessing fine-scale temperature responses. The results suggest that 1 °C increments do not uniformly affect lactic acid bacterial communities but can produce measurable compositional shifts under specific thermal conditions. These findings may be relevant for starter culture handling and fermentation process control, where precise temperature regulation can contribute to microbial stability and product consistency.

## 5. Conclusions

Precise Peltier-controlled incubation revealed that lactic acid bacterial community responses to 1 °C temperature differences differed between the cold (3–5 °C) and warm (36–38 °C) temperature conditions tested. Community composition remained stable at 3–5 °C but showed measurable changes within 36–38 °C, whereas viable counts of a representative *L. fermentum* culture and broad enzyme activity profiles showed limited variation. These findings support the usefulness of fine-scale temperature screening for evaluating microbial community stability in starter cultures. Future studies incorporating intermediate temperature points, strain-resolved analyses, and metabolite profiling will be valuable for establishing a continuous temperature–response relationship and for elucidating the functional and sensory consequences of these fine-scale, temperature-dependent community shifts.

## Figures and Tables

**Table 1:** Effect of incubation temperature (36–38 °C) on viable cell counts of *Limosilactobacillus fermentum*.

Temperature (°C)	Viable Cell Count (log CFU/mL)
36	9.03 ± 0.07
37	9.05 ± 0.04
38	9.05 ± 0.01

Values are expressed as mean ± standard deviation (SD) of triplicate experiments.

**Table 2:** Enzymatic activities of bacterial starter cultures incubated at 37 and 38 °C as determined using the API ZYM kit. Scores range from 0 to 5 based on color intensity. Scores of ≥3 were considered positive.

No.	Enzymes	37 °C/48 h	38 °C/48 h
1	Control	0	0
2	Alkaline phosphatase	1	1
3	Esterase	2	2
4	Esterase lipase	2	2
5	Lipase	1	1
6	Leucine arylamidase	5	5
7	Valine arylamidase	4	4
8	Cystine arylamidase	2	2
9	Trypsin	1	1
10	α-Chymotrypsin	0	0
11	Acid phosphatase	3	3
12	Naphthol-AS-BI-phosphohydrolase	3	3
13	α-Galactosidase	4	4
14	β-Galactosidase	0	0
15	β-Glucuronidase	3	3
16	α-Glucosidase	4	4
17	β-Glucosidase	5	5
18	N-acetyl-β-glucosaminidase	2	2
19	α-Mannosidase	1	1
20	α-Fucosidase	0	0

## Data Availability

The data supporting the findings of this study are included in the article. The raw 16S rRNA gene sequencing reads generated in this study have been deposited in the NCBI Sequence Read Archive (SRA) under BioProject accession number PRJNA1475241.
